# A VP26-mNeonGreen Capsid Fusion HSV-2 Mutant Reactivates from Viral Latency in the Guinea Pig Genital Model with Normal Kinetics

**DOI:** 10.3390/v10050246

**Published:** 2018-05-08

**Authors:** Julianna R. Pieknik, Andrea S. Bertke, Shuang Tang, Philip R. Krause

**Affiliations:** 1 Uniformed Services University of the Health Sciences, Bethesda, MD 20814, USA; julianna.pieknik@fda.hhs.gov; 2 Center for Biologics Evaluation and Research, Food and Drug Administration, Silver Spring, MD 20993, USA; Shuang.Tang@fda.hhs.gov; 3 Department of Population Health Sciences, Virginia Polytechnic Institute and State University, Blacksburg, VA 24061, USA; asbertke@vt.edu

**Keywords:** HSV-2, reactivation, guinea pig, fluorescent HSV-2, mNeonGreen, vaginal infection model

## Abstract

Fluorescent herpes simplex viruses (HSV) are invaluable tools for localizing virus in cells, permitting visualization of capsid trafficking and enhancing neuroanatomical research. Fluorescent viruses can also be used to study virus kinetics and reactivation in vivo. Such studies would be facilitated by fluorescent herpes simplex virus recombinants that exhibit wild-type kinetics of replication and reactivation and that are genetically stable. We engineered an HSV-2 strain expressing the fluorescent mNeonGreen protein as a fusion with the VP26 capsid protein. This virus has normal replication and in vivo recurrence phenotypes, providing an essential improved tool for further study of HSV-2 infection.

## 1. Introduction

Fluorescent alphaherpesviruses are important tools in neuroanatomical research, because fluorescent virus can cross synapses, aiding in mapping of neuronal pathways. They also allow for studies of virus assembly, maturation, and transport. Herpes simplex virus 1 (HSV-1) recombinants bearing fluorescent protein-tagged capsid protein (VP26) have proved particularly useful, due to the large number of fluorescent molecules per virion [1,2,3,4,5,6,7,8]. Previously described fluorescent HSV-1 mutants have used the dimeric green fluorescent protein (GFP), the dimeric yellow fluorescent protein (YFP), or the enhanced GFP, as well as monomeric versions of GFP, red fluorescent protein (RFP), and other fluorescent proteins (FPs) [9,10].

Efforts to optimize fluorescent viruses have focused on HSV-1 in which fluorescent proteins have been inserted into the N-terminal region of VP26. These viruses are infectious, but nonetheless have shown important differences from wild-type virus [2,3,4,6,7,10,11,12]. Dimerization of GFP and YFP, leading to nuclear aggregates, has been a persistent challenge [2,3,7,10]. Previous work examined the utility of replacing dimeric fluorescent proteins with monomeric ones, which reduced but did not eliminate aggregates, and was still associated with replication defects that manifested as diminished plaque size [4,6,10,12]. Some of these previous constructs also reverted to a non-fluorescent virus, likely due to competition from non-fluorescent mutants that arise during replication in culture [10,11,13].

Fluorescent viruses have the potential for aiding in study of HSV latency and reactivation, potentially making it easier to identify loci of viral reactivation or of incipient recurrent lesions. Studies of viral reactivation from neuronal latency are limited because no fluorescent HSV mutant has been shown to reactivate *in vivo* with wild-type kinetics. A previously described HSV-2 that expresses a VP26-eGFP fusion protein is genetically stable and has been useful for *in vitro* studies, but does not reactivate with normal kinetics *in vivo* [14,15,16].

Construction of a fluorescent HSV-2 that replicates and reactivates with normal kinetics in vivo would enable use of the female guinea pig genital model, in which virus reactivates spontaneously to cause recurrent lesions. We based an HSV-2 small capsid protein (VP26) fusion on the most successful HSV-1 and HSV-2 constructs, which employ N-terminal VP26 fusions with monomeric fluorescent proteins. Small deletions in the N-terminus of HSV-1 VP26 appeared necessary for optimal fusion, so we engineered a corresponding deletion into HSV-2. We also used a recently described monomeric fluorescent protein derived from *Branchiostoma lanceolatum* that is three times brighter than eGFP, mNeonGreen, which has not been previously used in HSV capsid fusions [17]. Using the guinea pig as a well-established spontaneous reactivation model and a novel, monomeric fluorescing virus with an optimized design, we have tested the value of a newly generated tool and a novel, bright fluorescent protein, which has potential use in other viruses or protein fusions. Ideally, a capsid-modified strain would have a similar morphology to wild-type strains, replicate with similar kinetics to the parental strain, fluoresce brilliantly enough to visualize with available microscopy, create similar cytopathic effects *in vitro*, cause similar pathology *in vivo*, and reactivate spontaneously from latency.

## 2. Materials and Methods 

Virus strains and stock production. HSV-2 Strain 333 was originally obtained from Gary Hayward (Johns Hopkins University, Baltimore, MD, USA). mNeonGreen was obtained from Allele Biotech (San Diego, CA, USA). Plasmid pUL35NeGr, expressing mNeonGreen as a *UL35* (encoding VP26) fusion, was constructed with GeneArt Seamless Cloning per manufacturer’s instructions. Briefly, flanking regions of *UL35* sequence (with codons 1–7 deleted from the region downstream of the intended NeonGreen insertion), pUC19L, and mNeonGreen were PCR-amplified with the High GC Fidelity Kit (Qiagen, Valencia, CA, USA) and overlapping primers. The overlapping primers used were: pUC19L (Forward: GAGTCGACGGCATGCAAGCTTGG, Reverse: GAACGCGTGTACCGAGCTCGAATT); HSV-2 upstream flanking region (Forward: CTCGGTACACGCGTTCGAGGGTC, Reverse: CTCACCATCGGGACCTTGGGTCG), mNeonGreen (Forward: AGGTCCCGATGGTGAGCAAGGGC, Reverse: CTGGGGCGCTTGTACAGCTCGTCC), dUL35 (Forward: TGTACAAGCGCCCCAGCACCATT, Reverse: TGCATGCCGTCGACTCCGCGCCC). Equal quantities of the purified fragments were then recombined into a single plasmid and transformed into DH10B cells. The constructed plasmid (pUL35NeGr) was verified by capillary sequencing (Core Facility, CBER, FDA, Silver Spring, MD, USA). The recombinant virus, which we named Nedel, was then generated in Vero cells (ATCC^®^ CCL-81™) by homologous recombination after transfecting with Superfect Transfection Reagent (Qiagen, Valencia, CA, USA), pUL35NeGr, and full length HSV-2 DNA. After plaque purification to homogeneity, the mutant virus was plaque purified three additional times.

Western Blot. Monolayers of Vero cells were infected with HSV-2 strain 333 or Nedel at a multiplicity of infection (MOI) of 10. Protein was extracted with Laemmli Buffer and separated using sodium dodecyl sulfate-polyacrylamide gel electrophoresis (SDS-PAGE) using Nu-Page 4–12% Bis-Tris gels (Invitrogen, Carlsbad, CA, USA) and transferred to nitrocellulose membranes (iBlot™ Transfer Stack, nitrocellulose, mini, Thermo Fisher Scientific, Waltham, MA, USA). Membranes were incubated with a rabbit peptide antibody to the C-terminus of VP26 (95RRTYSPFVVREPSTPGTP112), a generous gift of Prashant Desai, at a 1:500 dilution for 1 h at RT, horseradish peroxidase-linked secondary anti-rabbit antibodies (GE Healthcare, Cincinnati, OH, USA) were used at a 1:2000 dilution for 1 h at RT and detected by chemiluminescence (ECL) reagent (GE Healthcare). Magic marker protein standard (Invitrogen) was loaded into the first lane.

Growth Curve and Plaque size comparison. Growth of HSV-2 in Vero cells was characterized as previously described [18]. Briefly, Vero cells were infected with HSV-2 Strain 333 or Nedel at a MOI of 0.01, and total virus was collected from cells at 0, 6, 12, and 24 h post-infection (hpi). Strain 333 or Nedel was quantified by standard plaque assay on Vero cells. The area of plaques was determined by NIS Element software (v4.1) on a Nikon Eclipse Ti-E fluorescent microscope (Nikon, Tokyo, Japan) and the 6-point oval tool.

Primary adult neuron infection. Dorsal root ganglia were removed from 6 week old Swiss Webster mice, dissociated enzymatically and mechanically, and plated on Matrigel-coated Lab-Tek II chamber slides (Thermo Scientific, Waltham, MA, USA) as previously described [14]. Neurons were maintained in Neurobasal A media supplemented with B27, penicillin/streptomycin, Glutamax, neurotrophic factors, and mitotic inhibitors (Life Technologies, Carlsbad, CA, USA). Three days post-plating, neurons were inoculated with 30 MOI Nedel. For productive infections, neurons were fixed and immunolabeled for A5 (Fe-A5 supernatant, DSHB, Iowa City, IA, USA) or IB4 (isolectin B5-rho, Vector Laboratories, Burlingame, CA, USA) 9 h post-infection. For reactivation studies, infected neurons were maintained in 300 μM acyclovir to establish experimental latency for 7 days, followed by deprivation of neurotrophic factors to induce reactivation, and neurons were fixed and immunolabeled 9 h later. Neurons were counted and expressed as percentage of total or subpopulations of neurons productively infected. Reactivations were similarly counted, but subpopulations were represented as percentage of total reactivations that occurred in A5+ or IB4+ neurons. Neurons were imaged on inverted fluorescent Olympus IX73 microscope (Olympus Corporation, Tokyo, Japan).

Transmission Electron Microscopy. Epoxy-Resin Embedding was performed by Yamei Gao (FDA, CBER) as follows: Samples were fixed with 2.5% glutaraldehyde, post-fixed with 1% osmium tetroxide, stained with 2% uranyl acetate/water, dehydrated in a series of ethanol buffers, infiltrated with 1:1 (Epon12:P.O.), then infiltrated with 100% Epon12, and embedded in block molds. Ultrathin sections were cut by ultra-microtome (Leica EM UC7, Wetzlar, Germany) and were stained with 1% uranyl acetate/water. Samples were examined with the Zeiss L120 transmission electron microscope (Carl Zeiss AG, Oberkochen, Germany) and pictures were taken using the Gatan US 1000XP digital camera (Gatan, Inc., Pleasanton, CA, USA).

### In Vivo

Animals. Female Hartley Guinea Pigs (15 initially total per group, 150–200 g, Charles River Breeding Laboratories, Wilmington, MA, USA) were intravaginally inoculated with 1 × 10^6^ pfu HSV-2 (Strain 333 or Nedel) as previously described [19]. Animals that failed to produce any lesions or developed end-point criteria prior to day 14 were removed from analysis, resulting in 7 Strain 333-infected animals and 13 Nedel-infected animals. All animal experiments were performed under protocol number 1997-08 approved by the Center for Biologics Evaluation and Research Independent Animal Care and Use Committee (29 June 2015, CBER IACUC).

Replication In Vivo. Animals were evaluated daily for evidence of genital skin disease, urinary retention, hind limb paresis, paralysis, and death. Primary genital skin disease (Days 1–14 pi) was quantified by a lesion score system on a scale from 0 to 4 as follows: 0 for no disease, 1 for redness/swelling, 2 for one or two lesions, 3 for three to five lesions, and 4 for six or more lesions, coalescence of lesions or 3–5 lesions with neurologic symptoms [19]. 

Viral Shedding. Vaginal swabs were collected 5 times (Day 4, 7, 14, 21, 31 pi, *n* = 7 Strain 333, *n* = 13 Nedel). The presence of virus was determined by plaque assay on Vero cells and by fluorescence microscopy (Nikon Eclipse Ti-E) using Mouse anti-HSV-2 gH/gL (H2A269-100, Virusys, Taneytown, MD, USA), Mouse anti-HSV gB (HA056-100, Virusys), Mouse anti-HSV gD (HA025-100, Virusys), Goat anti-Mouse IgG (H + L) Highly Cross-Adsorbed Secondary Antibody, Alexa Fluor 546 (Thermo Fisher Scientific, catalog # A-11030, RRID AB_2534089).

Replication Ex Vivo. Dorsal Root Ganglia were harvested and cultured at 36 days post-infection from 5 guinea pigs intravaginally infected with Strain 333 or Nedel, as previously described [14]. Briefly, ganglia were digested in papain, collagenase, and dispase (Worthington Biochemical Corporation, Lakewood, NJ, USA) before mechanically triturating and plating on Matrigel-coated 8-well Lab-Tek II chamber slides (Thermo Scientific), followed by mechanical trituration with a pipette. Cultures were then fixed at 60 or 72 h post-plating for 5 min in 4% paraformaldehyde, gently rinsed in phosphate buffer saline (PBS) and immunolabelled as described below. Immunolabelled neuronal cultures were evaluated by fluorescence microscopy using a Nikon Eclipse Ti-E or a Zeiss LSM 710 upright confocal laser scanning microscope. 

Cryosectioning. Animals were cardiac-perfused with PBS followed by cardiac-perfusion with 4% paraformaldehyde. Tissues were dissected, rinsed in PBS and sucrose-protected overnight. Sections were embedded in OCT (TissueTek^®^, Sakura® Finetek, VWR catalog number 25608-9300, Radnor, PA, USA) and 10 µm sections were made with a cryostat (Leica, Wetzlar, Germany). Sections were permeabilized, blocked, and immunolabelled

Immunolabelling and microscopy of plaques, explanted ganglia, and cryosections. Antibodies used included: NeuroTrace™ 640/660 Deep-Red Fluorescent Nissl Stain (N21483 Thermo Fisher Scientific), Mouse anti-HSV-2 gH/gL (H2A269-100, Virusys, Taneytown, MD, USA), Mouse anti-HSV gB (HA056-100, Virusys), Mouse anti-HSV gD (Virusys, HA025-100), Goat anti-Mouse IgG (H + L) Highly Cross-Adsorbed Secondary Antibody, Alexa Fluor 546 (Thermo Fisher Scientific, catalog # A-11030, RRID AB_2534089). Post-permeabilization and blocking, samples were labelled with the primary antibody for 24 h at 4 °C, secondary antibody for 24 h at 4 °C, labelled with Nissl stain for 2 h at room temperature before DAPI staining. Cryosections also were treated with TrueBlack™ for 30 s in order to remove autofluorescence from lipofuscin that might otherwise potentially obscure our observations [20,21]. Nikon Eclipse Ti-E was used to take phase contrast and fluorescent images. A Zeiss LSM 710 upright confocal laser scanning microscope was used to provide more detailed fluorescent imaging. Uninfected and strain 333-infected ganglia were used to establish the maximum exposure times and gain that could be used before the emergence of autofluorescence. Single-stained explanted ganglia and cryosections were used to ensure that fluorescence was not bleeding over into other channels. Images of explanted ganglia were taken at 12, 24, and 48 h time points to establish a timeline of the emergence of fluorescence.

## 3. Results

### 3.1. In Vitro Characterization

**Figure 1:**
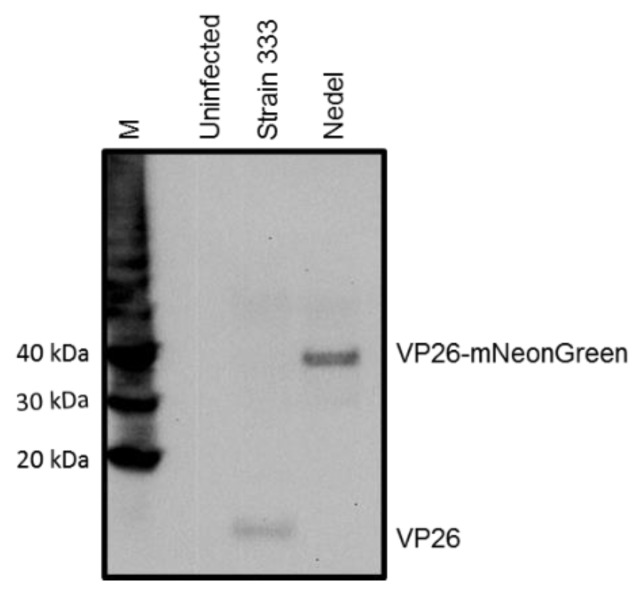
Western blot, visualized with anti-VP26 antibody, indicating that Nedel expresses VP26-mNeonGreen fusion protein. Vero cells were harvested 24 h after infection at an MOI of 10 with either Strain 333 or Nedel. Magic Marker protein standards (M), uninfected Vero cells, HSV-2 Strain 333-infected Vero cells, and Nedel-infected Vero cells are in the lanes left to right.

Our goal was to develop and characterize a fluorescent HSV-2 variant that could reactivate *in vivo* with wild type HSV-2 kinetics and maintain its fluorescence into the reactivation phase. One of the most successful previous approaches with HSV-1 and HSV-2 was chosen, but the fluorescent protein was replaced by the three-fold brighter monomeric mNeonGreen fluorescent protein as an N-terminal fusion to UL35 (VP26) with the first 21 base pairs of *UL35* deleted (corresponding to the first 7 codons), under control of the native *UL35* promoter. We constructed this HSV-2 mNeonGreen VP26 fusion by homologous recombination, and designated the recombinant as “Nedel”. In order to confirm that the fluorescent protein had been fused to the minor capsid protein, we performed a Western blot using an antibody to VP26 (Figure 1). The wild-type capsid protein is 12 kDa; when fused to the 24 kDa mNeonGreen, the fusion mutant VP26 migrates consistent with the expected size of 36 kDa.

**Figure 2:**
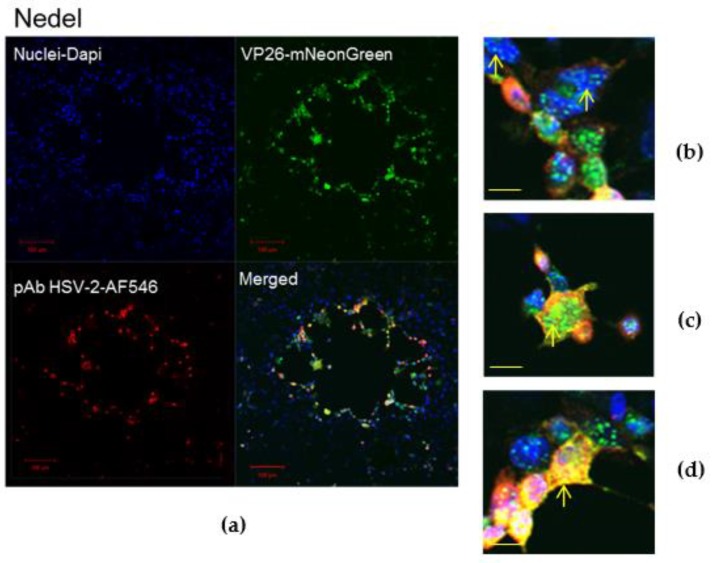

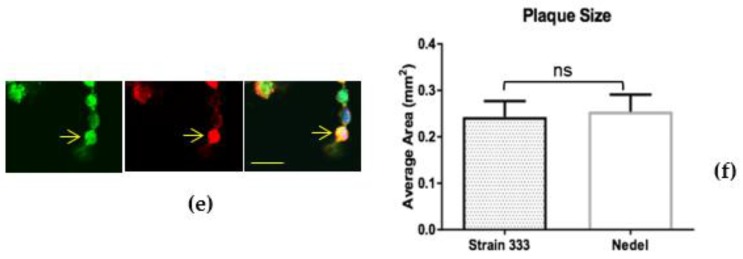
In Nedel-infected cells, mNeonGreen co-localizes with HSV antigen, and Nedel plaques are similar to wild-type. (**a**) Representative confocal image of a Nedel plaque on Vero cells. Nuclei (**upper left**) are stained with DAPI, mNeonGreen is detected (**upper right**) by fluorescence, and HSV-2 antigens are detected by immunofluorescent staining using a polyclonal HSV-2 antibody (**lower left**). Merged image is shown at **lower right**. Red bar = 100 µm. Enlarged images taken from the plaque shown in panel (**a**) illustrate fluorescence in the (**b**) nucleus, (**c**) cytoplasm, (**d**) and cell membrane, in some cases (**e**) obscuring the entire cell. Yellow arrow indicates location referenced in text; yellow bar = 15 µm. (**f**) Plaque size comparison, mean of 20 plaques, Error bars reflect SEM, Unpaired two-tailed *t* test *p* = 0.82.

The fluorescent capsid proteins in Nedel exhibited cellular distribution similar to that of HSV-2 glycoproteins, as assessed by immunolabelling in Vero cells (Figure 2a). Cytoplasmic fluorescent puncta, likely corresponding to assembled virions (Figure 2b), nuclear fluorescence (Figure 2c), and cell membrane-associated fluorescence (Figure 2d) were observed, frequently co-localizing with the HSV polyclonal antibodies, indicating that the fusion protein was incorporated into intranuclear capsids and mature virions. Occasionally, the fluorescence from the capsid and pAb overlapped in entire rounded cells without a more distinct fluorescence pattern (Figure 2e). In infected Vero cells (Figure 2f), there was no significant difference in plaque size between HSV-2 Strain 333 (mean plaque area = 0.24 mm^2^) and Nedel (mean plaque area = 0.25 mm^2^) (Unpaired t test, *p* = 0.82). 

**Figure 3:**
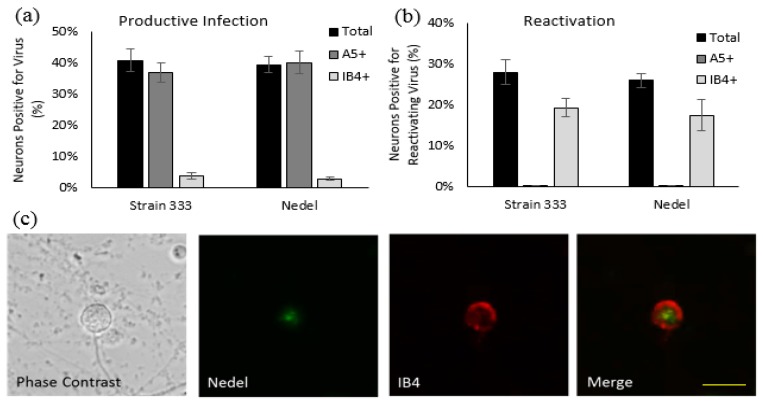
Nedel-infected cultured primary adult murine sensory neurons. (**a**) Productively infected neurons, quantified by counting total and immunolabeled A5+ and IB4+ subpopulations of neurons that contained mNeonGreen, expressed as a percentage of neurons counted. (**b**) Reactivating neurons, expressed as a percentage of total neurons reactivating and a percentage of total reactivations that occurred in A5+ and IB4+ neurons. (**c**) Representative image of Nedel reactivating in an IB4-labeled neuron, showing that Nedel reactivates in IB4+ sensory dorsal root ganglia neurons, as described previously for wild-type Strain 333 [16]; yellow bar = 15 µm.

To ensure that viral infection was not limited to Vero cells, we assessed the ability of Nedel to infect cultured primary adult murine DRG neurons. Nedel productively infected a similar percentage of total cultured DRG neurons compared to HSV-2 Strain 333 (39.5% and 40.9%, respectively), with preferential productive infection observed in A5+ neurons (Figure 3a), similar to previous reports [16]. Reactivation of Nedel also occurred comparable to Strain 333; approximately 70% of total reactivations occurred in IB4+ neurons, for both Strain 333 and Nedel, as opposed to less than 1% of total reactivations detected in A5+ neurons (Figure 3b). mNeonGreen expressed by Nedel during reactivation was clearly visualized within the immunolabeled primary adult cultured neurons (Figure 3c).

**Figure 4:**
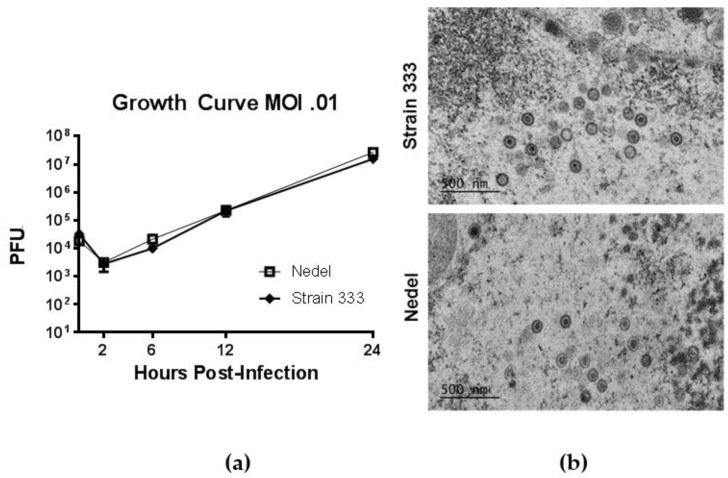
Replication of Nedel in vitro. (**a**) Growth Curves of Strain 333 and Nedel in Vero cells. Data points are the mean of three separate titrations and error bars reflect SEM. (**b**) Epoxy-resin-embedding transmission electron microscopic images of Strain 333 and Nedel in Vero cells.

In standard growth curves, to study in vitro replication of Nedel, monolayers of Vero cells were infected at a multiplicity of 0.01 PFU/cell with Strain 333 or Nedel, and virus was quantified by plaque assay from cultures harvested at indicated times post-infection (Figure 4a). Replication of Nedel was similar to that of Strain 333 over the course of 24 h (Figure 4a, Mann–Whitney U test, *p* = 0.90). By transmission electron microscopy (after epoxy-resin embedding) there was no significant difference between Nedel and Strain 333 in capsid size (~100–125 nm diameter), or virion morphology (Figure 4b). We did not observe proteinaceous aggregates or capsid aggregation, as has been reported for other fluorescent HSV [10]. 

### 3.2. In Vivo Characterization

**Figure 5:**
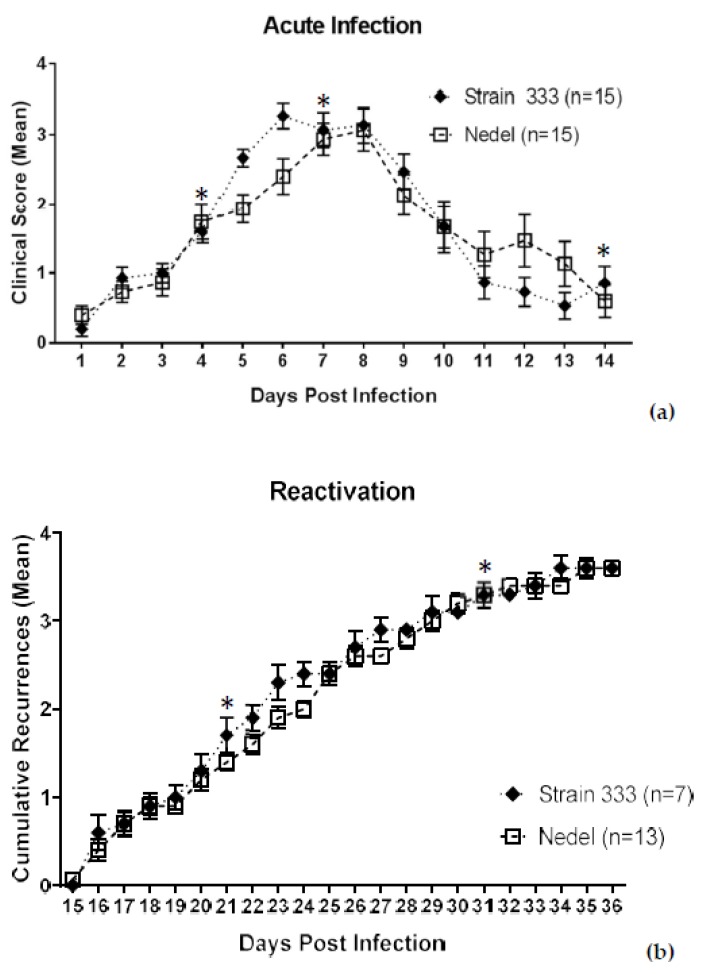
Characterization of Nedel in the guinea pig genital model of HSV infection. (**a**) Acute severity determined by genital lesion scoring on a scale from 0 to 4. Error bars represent SEM. Significance determined by two-way ANOVA, *p* = 0.79. (**b**) Mean cumulative number of recurrent genital skin lesions per guinea pig. Mean recurrence phenotype was determined by observing genital skin for new lesions. Error bars represent standard error of the mean. Two-way ANOVA, *p* = 0.93. Asterisks (*) above data points indicate days on which vaginal swabs with subsequent plaque assay were performed. On days 4 and 7, 13/13 Nedel-infected animals and 7/7 Strain 333-infected animals yielded 100% green and non-fluorescent plaques respectively. On day 14, 0% animals shed detectable virus. On both days 21 and 31, 2/13 of the Nedel-infected and 1/7 Strain 333-infected animals yielded 100% green plaques and 100% non-green plaques, respectively.

The ability of Nedel to infect adult female guinea pigs via the genital tract was also assessed. Among 15 Strain 333-infected animals, 7 developed bilateral hind-limb paralysis (requiring euthanasia per the animal protocol) and 1 showed no apparent acute infection. Of the remaining 7 showing evidence of acute infection, five developed hind-limb paresis (HLP), urinary retention, or unilateral paralysis. In the Nedel-infected group, two of the 15 animals were euthanized for bilateral transient hind-limb paralysis and 3 additional animals showed one or more of the other neurological symptoms. Thus, 12/15 Strain 333-infected animals developed neurological symptoms, as compared with 5/15 in the Nedel-infected group. While less neurovirulent *in vivo* than the Strain 333 virus, results in the Nedel group were similar to the percentage typically observed in animals infected with MS strain (with reported HLP rates of 12–20%) [22,23]. Despite the difference in neurovirulence, the infection with Nedel resulted in acute-phase lesions similar to those of Strain 333 (AUCs for Nedel and Strain 333 were 22.5 and 21.8, respectively, when all 15 animals were analyzed, and 22.4 and 22.1, respectively, when only the animals that were observed for the full 14 days were included in the analysis), although the number of days to peak mean lesion score lagged by ~2 days (Figure 5a. Acute mean lesion score statistical comparison by two-way ANOVA, *p* = 0.87). Vaginal swabs from 13/13 of Nedel-infected animals yielded 100% green fluorescent plaques on days 4 and 7 post-inoculation. On day 14, none of the swabs were positive for virus, signaling the end of the acute infection. Recurrence phenotype was determined by observing genital skin for new lesions each day (Days 15–36 pi) (Figure 5b). Mean cumulative recurrences were nearly identical between Nedel and Strain 333-infected animals, each with 3.6 recurrences over the course of 21 days (Two-way ANOVA, *p* = 0.93). Vaginal swabs collected on both day 21 and 31 post-infection were positive from 15% (2/13) of the Nedel-infected animals, similar to 14% (1/7) of Strain 333-infected animals that also shed infectious virus on each of those days. All plaques from positive swabs from the Nedel group were uniformly fluorescent green. With one exception (on day 21 in the Nedel group), all of these culture-positive animals had recurrences at the time of swabbing. 

### 3.3. Ex Vivo Characterization

**Figure 6:**
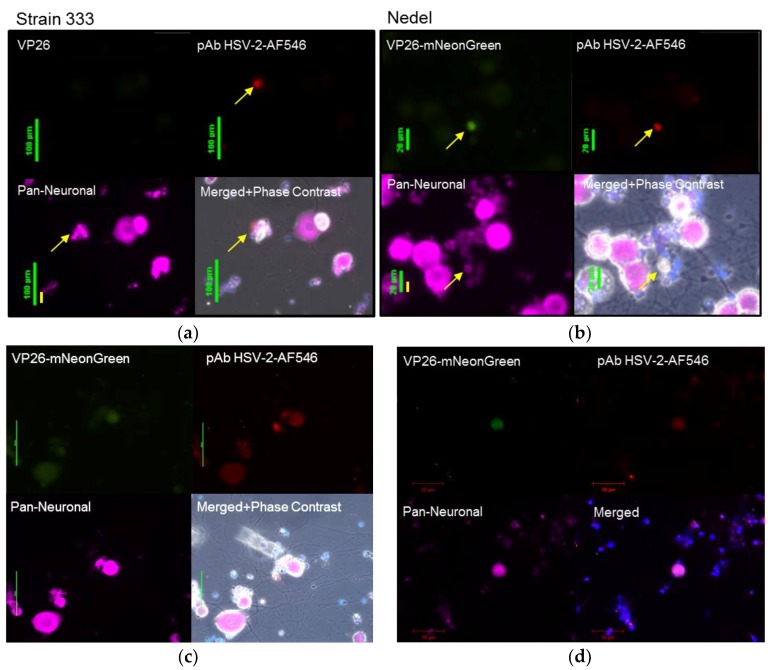
Ex vivo reactivation from explanted sacral dorsal root ganglia. Fluorescent images of enzymatically dissociated sensory neurons from guinea pigs 36 days post-infection and 72 h post-plating with either (**a**) Strain 333, green bar = 100 µm (**b**) Nedel, green bar = 20 µm, yellow bar = 10 µm. **Upper left**: Detection of mNeonGreen fluorescence (Strain 333 shows no fluorescence), **Upper right**: immunofluorescence using pAb HSV-2-AF546, **Lower left**: Pan-Neuronal stain, **Lower right** quadrant: Merged. Yellow arrow indicates reactivating neuron. (**c**) Explant with phase contrast, pAb HSV-2-AF546, DAPI, and a Pan-Neuronal stain, green bar = 50 µm (**d**) Confocal image of explant with staining strategy used in (**a**–**c**) without phase contrast, red bar = 50 µm.

Viral reactivation was also assessed *ex vivo* from infected animals euthanized on day 36 post-infection. Sacral dorsal root ganglia (DRG) were dissected, enzymatically digested and plated on Matrigel-coated chamber slides. In this model, the axotomy alone stimulates *ex vivo* reactivation [24,25]. Slides were then observed for fluorescence. Fluorescent neurons were first observed in cultures at ~60 h post-plating, with maximum fluorescence observed at 72 h post-plating. Cultures were fixed and stained to label neurons and HSV-2 antigen (Figure 6a,b). The neurons from Nedel-infected animals that exhibited green fluorescence also stained positively for non-capsid HSV-2 antigen, further supporting the idea that Nedel is capable of establishing latency in these neurons and of induced reactivation *ex vivo* after axotomy. Higher fluorescence intensity was observed in small neurons with altered morphology, suggestive of cytopathic effect from a recent reactivation. Double instead of triple staining of cultures (Pan-Neuronal and DAPI) allowed the analysis of more neurons per field of view (Figure 6c), also showing co-localization of green fluorescence and explanted neurons from Nedel-infected animals. Using confocal microscopy, we also observed similar co-localization of green fluorescence (representing the Nedel capsid protein) and red fluorescence (representing HSV glycoproteins) within neurons (Figure 6d).

### 3.4. In Vivo Cryosections

**Figure 7:**
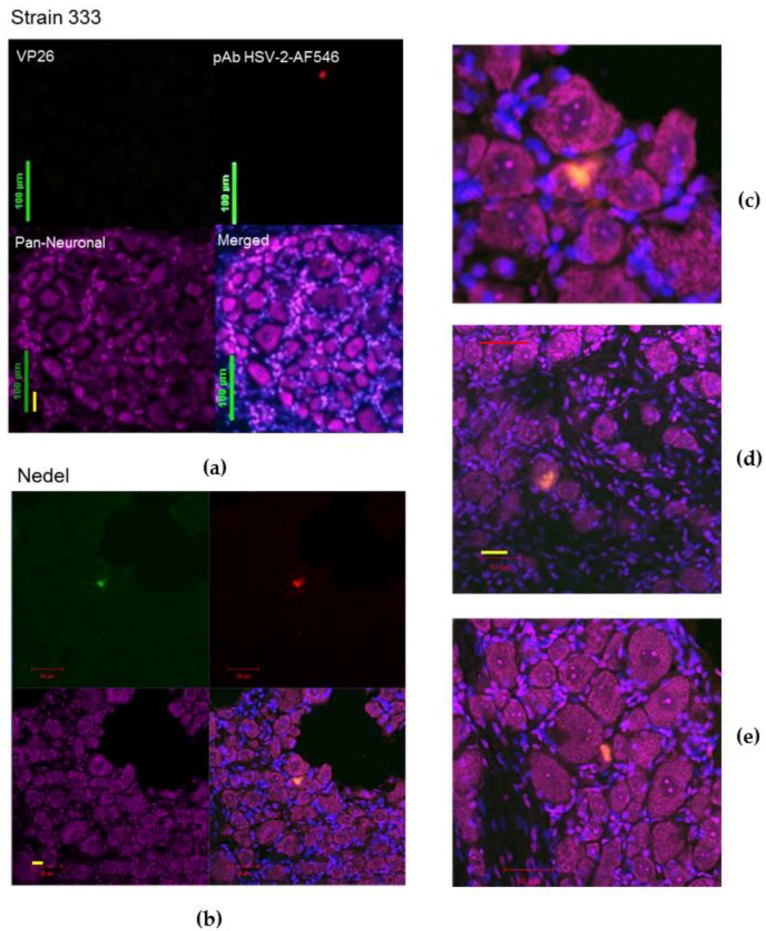
In Vivo Reactivation. Representative confocal microscopic images of immunolabelled cryosections of guinea pig sacral dorsal root ganglia infected with (**a**) Strain 333, green bar = 100 µm, yellow bar = 30 µm (**b**) Nedel. **Upper left**: Detection of mNeonGreen fluorescence, **Upper right**: immunofluorescence using pAb HSV-2-AF546, **Lower left**: Pan-neuronal stain, **Lower right**: Merged, (**c**) enlarged (merged) image of reactivation shown in (**b**); red bar = 25 µm. (**d**) Enlarged (merged) image of different reactivation, red bar = 50 µm, yellow bar = 33 µm. (**e**) Enlarged (merged) image of another reactivation.

To study the ability of the mutant virus Nedel to spontaneously reactivate in vivo from neurons, animals were cardiac-perfused and cryosections of the sacral DRG were made. Neurons were labelled with Nissl and sections with green fluorescence were confirmed to be HSV-2 positive by immunofluorescent labelling (Figure 7a–e). Because the control Strain 333 sections (e.g., as in Figure 7a) were selected at random for staining (while Nedel sections were selected by fluorescence), only one neuron (on a single slide out of twenty stained) was identified that stained positively with the HSV-2 pAb. The Nedel *in vivo*-infected neurons exhibited co-localization of intense fluorescence (green, upper left quadrant) and HSV-2 pAb (red, upper right quadrant) within the nuclei and cytoplasm of neurons (merged, bottom right quadrant). This pattern was consistently observed in sections from Nedel-infected animals that exhibited fluorescence from within neurons (Figure 7c–e). Thus, fluorescence in Nedel-infected ganglia indicated viral reactivation in these neurons.

## 4. Discussion

We describe the construction and evaluation of a fluorescent HSV-2 variant. Unlike previously described fluorescent HSV, this virus is genetically stable, has normal morphology without aggregation, replicates in vitro and in vivo with normal kinetics, and has a wild-type spontaneous recurrence phenotype in vivo. 

We attribute the successful construction of this virus to properties of the mNeonGreen fluorescent protein, which is much brighter than other fluorescent proteins and has no known tendency to aggregate. Although we did not formally analyze the number of incorporated VP26 molecules in Nedel, this likely allows for more normal VP26 interactions within the rest of the capsid and prevents aggregation of capsid proteins before assembly and of the capsids themselves before envelopment. Monomeric NeonGreen emits a yellow-green fluorescence (λem,max = 517 nm and λex,max = 506 nm), which may limit its use in highly autofluorescent tissues like the skin. 

While Nedel exhibited wild-type kinetics of acute and recurrent infection of guinea pigs, we noted reduced neurovirulence during the acute infection when an inoculum of 10^6^ pfu was used. We did not construct a rescuant for Nedel since the recombinant virus otherwise behaved as wild type HSV-2 Strain 333, so we do not know if this reduced neurovirulence is due to the VP26 mutation, or another unintentionally introduced mutation. The reduced neurovirulence observed with Nedel is in the range observed with other HSV-2 strains, and did not impact establishment of latency or viral reactivation. 

We examined the capacity of Nedel to reactivate in four different ways. *In vitro*, Nedel reactivated preferentially in primary adult cultured sensory neurons at a rate similar to wild-type virus. *In vivo* spontaneous reactivation evaluated the ability of reactivations to give rise to external recurrent lesions. *Ex vivo* reactivation after axotomy assessed the ability of the virus to reactivate from within individual neurons after a strong stimulus, and typically led to altered morphology of neurons harboring reactivated virus. Identification of green fluorescence in cryosections indicated the ability of the virus to spontaneously reactivate from these neurons *in vivo*. The ability to scan unstained sections for green fluorescence greatly simplified the identification of these loci of viral reactivation. We did not identify morphological changes in the neurons harboring reactivating virus *in vivo*, which is consistent with a previous report [24]. 

Nedel is the first described HSV-2 fluorescent strain with a normal reactivation phenotype. We intend to use this virus to study early reactivation events in vivo in more detail than has previously been possible. Inclusion of the mNeonGreen protein may also enhance the utility of fluorescent fusions of other herpesviruses and allow observation of infrequent or minimally produced proteins.
